# The Transport of Invalids by Rail

**Published:** 1896-10-31

**Authors:** 


### The Transport of Invalids by Rail.

At the present time of year, when in many households where sickness reigns the question whether it is possible to remove the invalid has become an urgent one, we think it well to draw attention to the great facilities offered by the railway companies for this purpose. It is the fashion with many people to say that if an invalid cannot go abroad, to Egypt, or to high altitudes, or to some place that may have come into vogue for his particular complaint, he had better stay by his own fireside. No doubt for people of small means this is often very true. Nothing is more wretched than the lot of the poor invalid in lodgings far from home, who has to look at every shilling, and consider the cost of each fire that is lit in his bedroom, and of each delicacy by which his appetite is tempted. But where a little money can be spent with a clear conscience, and especially where it is possible to remove the home and to surround the patient with his home belongings, there are many places along our English South Coast where patients can live in safety through the winter who would die, or, at least, would only just hold their own, in parts of the country less favourably situated. To many, then, to be transported safely to such havens means almost life, and both trouble and money are well spent in attaining such a result. A correspondent writing to us on this subject says: "I think you might usefully draw the attention of your readers to the possibility of transporting patients by rail for long distances, even when they are very seriously ill. A few days ago it became an extremely urgent matter that a patient suffering from phthisis, who had very large cavities, steadily increasing expectoration, which was becoming offensive, night sweats, an evening temperature ranging between 100 deg. and 102 deg., and a very weakened heart muscle, should be removed from a large Yorkshire town to Bournemouth. The course we adopted was simple and very successful. A horse ambulance, which belongs to the corporation for the use of the town, was provided by the chief of the fire brigade, who has entire charge of it, and brought to the door of the house. The patient was carried downstairs in a carrying-chair and placed upon a stretcher in the hall, and, after being well wrapped up, was then lifted into the ambulance and quietly driven to the railway station in company with his doctor and nurse. There the patient was carried from the ambulance to an invalid-carriage provided by the Midland Railway Company, in which was a couch suspended from spiral springs attached to the roof; relays of hot-water tins were arranged for at various places on the route; a special engine was provided by which the carriage was transferred from the west to the east station at Bournemouth; the manager of the Burlington Hotel was good enough to provide an omnibus in which the patient could lie down, and a couple of porters by whom, on arriving at his house, he could be carried from the omnibus straight up into his bedroom. The experiment was successful in a wonderful degree. The patient was certainly very exhausted on arrival, but he soon revived. He was at once able to keep his windows open, his appetite returned, and for the first time for many weeks his temperature was only 99 deg. in the evening. I will not speculate as to the future, but, at any rate, the transport, which had seemed a most risky adventure, proved a grand success—thanks to the exactitude with which the arrangements were carried out by the railway companies and others." This is an interesting bit of clinical experience, showing how by care and forethought patients may be transported even when very ill. We would, however, at the same time earnestly dissuade anyone from attempting such an adventure without taking full precautions and considering every possible contingency beforehand, for it is clear that the slightest breakdown in the arrangements might lead to serious difficulties. Everything must be thought of and be personally looked into. One person should be responsible, and nothing should be taken on trust. Food must be provided in proper form, and it should be such as the patient is accustomed to having; arrangements must be made for heating it; blankets and plenty of pillows must be taken; those in charge of the arrangements must be careful not to leave matters to some unauthorised railway clerk, but to get everything in black and white from the central office of the railway company as to carriage, shunting engines, and hot-water tins; the local transport at each end must be entrusted to reliable people; and, finally, the patient should be accompanied by both a doctor and a nurse. With such precautions very feeble folk can be carried long distances by rail, but no precautions can entirely obviate the fatigue of travel. Even the noise alone is tiring, and it is impossible by any mechanical contrivance entirely to do away with vibration. Probably slinging is the best plan for the latter purpose, but unfortunately by it the effects of oscillation are almost increased. Lastly, it is worth bearing in mind how largely the fatigue of travelling is aggravated by the visual weariness of watching the fleeting landscape, and how it may be lessened by keeping the blinds tightly drawn.

We need not enter into the medicinal treatment of the various contingencies which may possibly arise. It may be well, however, to recall to mind the efficacy of bromides in allaying the irritability produced by travel, and in lessening the nervous expectancy and interest in the journey, which itself is no mean factor in the production of exhaustion.

